# Metabolic alterations within the primary visual cortex in blind patients with end-stage glaucoma: a proton magnetic resonance spectroscopy study

**DOI:** 10.3389/fcell.2025.1590460

**Published:** 2025-06-27

**Authors:** Wenqing Zhu, Linying Guo, Wenwen Chen, Tingting Liu, Xinghuai Sun

**Affiliations:** ^1^ Department of Ophthalmology and Visual Science, Eye and ENT Hospital, Shanghai Medical College, Fudan University, Shanghai, China; ^2^ Key Laboratory of Myopia and Related Eye Diseases, NHC, Fudan University, Shanghai, China; ^3^ Key Laboratory of Myopia and Related Eye Diseases, Chinese Academy of Medical Sciences, Fudan University, Shanghai, China; ^4^ Departments of Radiology, Eye and ENT Hospital, Shanghai Medical College, Fudan University, Shanghai, China; ^5^ State Key Laboratory of Medical Neurobiology and MOE Frontiers Center for Brain Science, Institutes of Brain Science, Fudan University, Shanghai, China

**Keywords:** glaucoma, magnetic resonance spectroscopy, primary visual cortex, cortical plasticity, blindness

## Abstract

**Introduction:**

Glaucoma, a leading cause of irreversible blindness worldwide, imposes a devastating burden on over 11 million end-stage patients through permanent vision loss. Despite this profound disability, the neurochemical basis of preserved cortical plasticity remains unclear, compounded by the challenge of recruiting this vulnerable population for advanced neuroimaging studies.

**Methods:**

We conducted single-voxel proton magnetic resonance spectroscopy (1H-MRS) in 11 blind patients with end-stage primary open-angle glaucoma (POAG) and 11 normal controls to characterize metabolic alterations in the primary visual cortex (V1) and their relationship to residual retinal function.

**Results:**

Glutamate-glutamine complex (Glx), N-acetylaspartate (NAA), choline (Cho), and myo-inositol (Ins) ratios relative to creatine (Cr) were quantified, revealing significantly elevated Glx/Cr in POAG (95% CI: 0.09 ∼ 0.63, P = 0.011), while NAA/Cr, Cho/Cr, and Ins/Cr remained stable (P > 0.05). Notably, the Glx/Cr ratio correlated significantly with the N1-wave latency of mfERG (ρ = -0.676, P = 0.022), independent of other clinical parameters.

**Discussion:**

These findings demonstrate glutamate hyperactivity coexisting with preserved neuronal and osmotic homeostasis in the V1 of end-stage POAG patients, suggesting adaptive neuroglial compensation. The correlation between Glx/Cr ratios and mfERG responses indicates persistent retinocortical signaling despite blindness, highlighting the potential of 1H-MRS as a valuable tool for assessing cortical plasticity in advanced glaucoma rehabilitation.

## Introduction

Glaucoma, a neurodegenerative disorder affecting the entire visual pathway, currently impacts over 79 million individuals globally, of whom more than 11 million will progress to bilateral blindness (Stevens et al., 2013; Tham et al., 2014; Quigley and Broman, 2006). While traditionally viewed as an ocular disease, accumulating evidence positions glaucoma within the spectrum of central nervous system neurodegeneration, characterized by progressive anteroposterior visual pathway deterioration (Jonas et al., 2017).

Advanced magnetic resonance imaging (MRI) techniques have uncovered structural and functional cortical alterations in glaucoma, including volumetric fluctuations, cerebral blood flow reductions, and abnormal visual cortical activation patterns (Wang et al., 2016; Harris et al., 2013; Duncan et al., 2012; Duncan et al., 2007; Qing et al., 2010). Complementary immunohistochemical evidence further identifies neurochemical perturbations within the primary visual cortex (V1), such as dysregulated glutamate receptor expression and astrocytic hypertrophy, implicating cortical plasticity as a modulator of neurodegeneration progression (Lam et al., 2003). Despite these advances, conventional neuroimaging remains limited in resolving dynamic metabolic substrates underlying neuroadaptive processes.

Proton magnetic resonance spectroscopy (^1^H-MRS) noninvasively quantifies neurometabolites by detecting characteristic resonance frequencies of hydrogen atoms (^1^H) in brain tissue. When placed in a strong magnetic field, protons in different molecular environments resonate at distinct frequencies, allowing discrimination of metabolites such as: glutamate-glutamine complex (Glx, reflecting excitatory neurotransmission and astrocytic recycling), N-acetylaspartate (NAA, a surrogate for neuronal density and mitochondrial function), choline (Cho, indicative of membrane phospholipid turnover), myo-inositol (Ins, a gliosis and osmoregulation marker), and creatine (Cr, serving as an internal reference for metabolic normalization). This technique has been validated for reproducibility and accuracy against biochemical assays in visual pathway disorders, establishing its reliability for mapping neurochemical alterations (Ozsoy et al., 2012; Zhang et al., 2013; Wu et al., 2013). Prior ^1^H-MRS studies demonstrate spatially and temporally distinct metabolic responses in glaucoma: Anterior visual structures (e.g., lateral geniculate nucleus) exhibit decreased NAA/Cr and Cho/Cr (Zhang et al., 2013), reflecting irreversible neuronal loss and membrane degeneration secondary to retrograde neurodegeneration. Conversely, the posterior visual cortex shows early-stage adaptations, including elevated Glx/Cr and reduced Ins/Cr, indicating glutamate-mediated plasticity and impaired osmoregulation (Guo et al., 2018). Bernabeu et al. demonstrated Ins elevation in the occipital cortex of blind subjects with retinopathy or optic neuritis (Bernabeu et al., 2009). **Contrasting with this,** we postulated that chronic intraocular pressure stress in glaucoma may drive distinct glial responses, potentially altering Ins **dynamics in V1.** End-stage glaucoma represents a critical phase where irreversible blindness occurs, yet cortical plasticity mechanisms remain poorly understood. Studying neurochemical changes in these patients is vital because identifying preserved metabolic pathways reveals potential neuroprotective or compensatory mechanisms that could be harnessed for rehabilitation. Moreover, the correlation between neurochemical signatures in V1 and residual retinal function challenges the dogma of complete visual pathway disconnection in blindness, suggesting intact retinocortical signaling that could inform strategies to optimize residual vision.

This study investigates two underexplored dimensions: (1) whether metabolic alterations persist within V1 in blind patients with end-stage glaucoma, and (2) how residual retinal electrophysiology interacts with cortical metabolic profiles in these patients, aiming to identify neurochemical signatures for future rehabilitation strategies.

## Materials and methods

### Participants

Eleven blind individuals with bilateral end-stage primary open-angle glaucoma (POAG) (2 females and nine males; mean age 33.8 ± 13.1 years, range 20–56 years) were recruited from the Ophthalmology Department of the Eye and Ear, Nose, and Throat (EENT) Hospital, Fudan University, Shanghai, China. The eligibility criteria were as follows.

1. Bilateral 10° central scotoma confirmed by Goldmann kinetic perimetry and microperimetry;
2. Best-corrected visual acuity (BCVA) ≤ logMAR 1.30 (Snellen <20/400);
3. Diagnosis of POAG by a glaucoma specialist and follow-up by a glaucoma medical team for more than 5 years;
4. Intraocular pressure (IOP) stabilization (≤21 mmHg) for more than 3 years;
5. Documented stability of the BCVA and visual field over the preceding 3 years;The normal control group (NC) recruited eleven healthy subjects (3 females and eight males; mean age 35.75 ± 12.5 years; range 22–56 years) who exhibited BCVA ≥ logMAR 0.0 (6/6), normal achromatic perimetry, and absence of ocular pathology/glaucoma family history. Exclusion criteria for all participants included claustrophobia, ferromagnetic implants, major psychiatric comorbidities, or structural brain anomalies.

The studies involving humans were approved by the Ethics Committee of the EENT Hospital, Fudan University. The studies were conducted in accordance with the local legislation and institutional requirements. The participants provided their written informed consent to participate in this study.

### Examination protocol

All bilateral end-stage POAG participants underwent systematic ophthalmic assessments utilizing standardized protocols. Visual acuity was quantified through the XK100 logarithmic low-vision chart (Wenzhou Xinkang Medical), followed by subjective refraction to establish BCVA. IOP measurements were obtained *via* Goldmann applanation tonometry (Haag-Streit), while structural evaluations included slit-lamp biomicroscopy and optic nerve/macular imaging using the non-mydriatic retinal camera (CR-DGi, Canon). Visual field characterization integrated Goldmann kinetic perimetry and Octopus 900 static threshold analysis (Haag-Streit), with absolute scotoma defined as non-detection of maximum stimulus (Size V, 1.72° visual angle). A retinal microperimeter (MP- 1, Nidek) was utilized to map the location of the preferred retinal locus (PRL) and to assess the threshold of the residual visual field. The retinal layer thickness in the temporal islet region was measured using frequency-domain optical coherence tomography (RTVue FD-OCT, Visionix). Electrophysiological profiling employed multifocal electroretinography (mfERG, Veris 6.0, Electro-Diagnostic Imaging) to record P/N1-wave parameters (amplitude/latency) in temporal islet regions. Normal controls received a routine ophthalmic examination, including slit-lamp examination, visual acuity, fundus photography, IOP, visual field, and refraction, with all procedures conducted by certified ophthalmologists.

### MRI data acquisition

All participants underwent MRI on a 3.0 T scanner (Verio; Siemens, Erlangen, Germany) equipped with a 32-channel head coil. Sagittal, axial, and coronal T2-weighted turbo spin-echo (TR/TE, 4,000/97 m) images were obtained to exclude the presence of organic lesions in the brain and to locate the voxel of interest for ^1^H-MRS. No participants required sedation. All subjects completed MRI scans without pharmacological intervention to avoid neurometabolic confounds.

**FIGURE 1:**
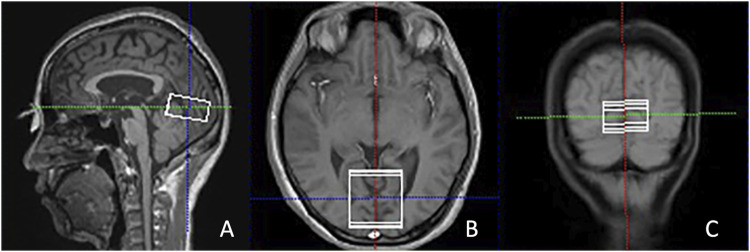
Illustration of the VOI placement in the primary visual cortex along the calcarine sulcus in the sagittal **(A)**, axial **(B)**, and coronal **(C)** views in T2-weighted images from a POAG patient. POAG indicates primary open-angle glaucoma; VOI, volume of interest.

Single-voxel ^1^H-MRS was subsequently conducted using the PRESS technique (TR/TE = 3,000/30 m, 80 acquisitions), with the automated placement of the voxel (40 × 40 × 20 mm) along the calcarine sulcus. Spectral optimization utilized iterative shimming procedures: initial automated B-field homogenization to achieve a full width at half maximum (FWHM) of ≤30, with additional manual refinements applied if the FWHM surpassed 30. Water suppression was performed. The volume of interest (VOI) placement was carefully directed to target the bilateral V1 by aligning anatomically with calcarine sulcus markers across perpendicular planes, ensuring extensive coverage of Brodmann area 17 parenchyma (Figure 1).

### MRS data processing

**FIGURE 2:**
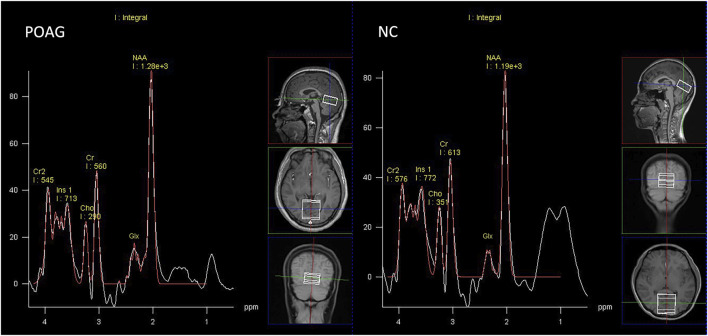
Representative proton spectra in the primary visual cortex acquired from a POAG patient and normal control.

The peak integrals of Glx, NAA, Cho, Ins, and Cr were measured using the spectroscopy toolkit within the Syngo MR software (Siemens, Erlangen, Germany). This was conducted following a series of processing steps, including residual water suppression, zero-filling expansion from 1,024 to 2048 data points, baseline adjustment, Fourier transformation, phase correction, and fitting of the spectral curves. The NAA spectral peak occurred at 2.02 ppm, while Glx was observed between 2.1 and 2.5 ppm. Ins exhibited a peak at 3.56 ppm, Cho at 3.22 ppm, and Cr at 3.03 ppm (as shown in Figure 2). The ratios of NAA to Cr, Cho to Cr, Glx to Cr, and Ins to Cr were automatically calculated.

### Statistical analysis

Statistical analyses were performed using SPSS 20.0 (IBM, Chicago, IL, United States). Demographic comparability between POAG and NC was verified by independent t-test (age) and χ^2^ tests (sex distribution). Metabolic ratios (Glx/Cr, NAA/Cr, Cho/Cr, Ins/Cr) underwent group-wise comparison through independent sample t-test with 95% confidence intervals. Bivariate Pearson correlations evaluated associations between MRS-derived metrics and clinical parameters (BCVA, visual field indices, retinal thickness, mfERG waveforms). A *P* value of less than 0.05 was considered to be statistically significant.

## Results

Demographic and clinical characteristics of the study cohorts are summarized in Table 1. No significant intergroup differences were observed in age (*P* = 0.831) or sex distribution (*P* = 1.000).

**TABLE 1:** Demographic features and clinical information of the participants.

Demographic features and clinical information	POAG group	NC group
No. Subjects	11	11
Gender (male/female)	9/2	8/3
Age (median, range), years	33.73 ± 13.07 (29, 20–56)	34.91 ± 12.51 (31, 22–56)
Intraocular Pressure (median, range), mmHg	18.25 ± 2.28 (18, 14–21)	16.50 ± 2.05 (16, 13–20)
Vertical cup-to-disc ratio (median, range)	0.95 ± 0.05 (0.90, 0.90–1.00)	0.35 ± 0.05 (0.30, 0.20–0.40)
Duration since initial POAG diagnosis (median, range), years	9.50 ± 2.15 (10, 7–15)	NA
History of anti-glaucoma surgery (no., %)	6 (54.55%)	NA
Ongoing topical anti-glaucoma therapy (no., %)	7 (63.64%)	NA

Abbreviations: POAG, primary open-angle glaucoma; NC, normal control; NA, not applicable.

**FIGURE 3:**
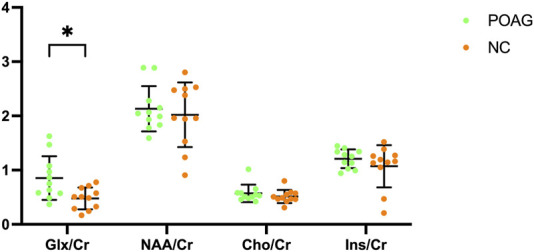
Boxplot illustrating Glx/Cr, NAA/Cr, Cho/Cr, and Ins/Cr for the POAG patients and normal controls within the bilateral primary visual cortex. Error bars represent standard deviation.

Metabolic profiling of the V1 revealed distinct neurochemical alterations in POAG patients (Table 2). The Glx/Cr was significantly elevated in the POAG cohort compared to the controls (0.84 ± 0.38 vs 0.48 ± 0.20, 95% CI 0.09–0.63, *P* = 0.011). In contrast, no group differences emerged in neuronal integrity markers (NAA/Cr: 2.13 ± 0.42 vs 2.02 ± 0.60, *P* = 0.618), membrane turnover indices (Cho/Cr: 0.57 ± 0.16 vs 0.51 ± 0.12, *P* = 0.347), or osmoregulatory metabolites (Ins/Cr: 1.21 ± 0.17 vs 1.07 ± 0.39, *P* = 0.297). The Glx/Cr, NAA/Cr, Cho/Cr, and Ins/Cr for the POAG patients and the normal controls are illustrated in the boxplot in Figure 3.

**TABLE 2:** Comparison of the metabolite ratios in the primary visual cortex between POAG patients and normal controls.

Metabolite ratios	POAG group	NC group	95% CI	t Value	*P* value
Glx/Cr	0.84 ± 0.38	0.48 ∼ 0.20	0.09∼0.63	2.783	0.011*
NAA/Cr	2.13 ± 0.42	2.02 ± 0.60	−0.35∼0.57	0.506	0.618
Cho/Cr	0.57 ± 0.16	0.51 ± 0.12	−0.07∼0.19	0.963	0.347
Ins/Cr	1.21 ± 0.17	1.07 ± 0.39	−0.13∼0.41	1.071	0.297

Values are given as mean ± SD.

Abbreviations: Glx, glutamate-glutamine complex; Cr, creatine; NAA, N-acetylaspartate; Cho, choline; Ins, myo-inositol; POAG, primary open-angle glaucoma; NC, normal control; CI, Confidence Interval.

*P < 0.05

**FIGURE 4:**
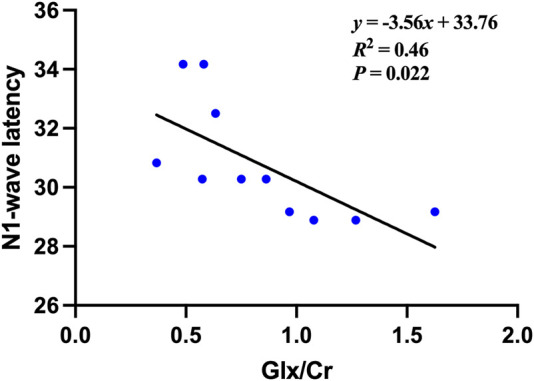
The Correlation between the Glx/Cr ratio and the N1-wave latencies of the multifocal electroretinography (mfERG) in the POAG patients.

Pearson correlation analysis demonstrated a significant relationship between Glx/Cr ratios and electrophysiological function in POAG patients. Among exploratory correlations, the strongest association was between Glx/Cr and mfERG N1 latency (ρ = −0.676, uncorrected *P* = 0.022, 95% CI [−0.89, −0.21]), though no associations were observed with structural parameters (retinal thickness), perceptual metrics (BCVA, visual field), or other clinical indices (Figure 4). The remaining metabolite ratios (NAA/Cr, Cho/Cr, and Ins/Cr) showed no significant correlations with any assessed functional or anatomical measures. The correlations between metabolites and clinical data in the POAG patients are shown in Table 3.

**TABLE 3:** Correlations between metabolites and clinical data in the POAG patients.

Metabolite ratios		N1-wave latency	N1-wave amplitude	P-wave latency	P-wave amplitude	BCVA	Visual field	Retinal thickness
Glx/Cr	Correlation coefficient	−0.676	−0.178	−0.516	0.067	0.067	0.006	0.093
	*P* Value	0.022*	0.601	0.104	0.845	0.844	0.985	0.785
NAA/Cr	Correlation coefficient	−0.551	−0.360	−0.230	0.335	−0.492	0.324	−0.044
	*P* Value	0.079	0.277	0.496	0.314	0.125	0.332	0.899
Cho/Cr	Correlation coefficient	−0.329	0.036	−0.267	−0.165	0.459	−0.291	−0.211
	*P* Value	0.323	0.916	0.427	0.628	0.155	0.386	0.534
Ins/Cr	Correlation coefficient	−0.383	−0.226	−0.230	0.159	0.016	−0.137	−0.365
	*P* Value	0.245	0.505	0.495	0.641	0.962	0.689	0.270

Abbreviations: Glx, glutamate-glutamine complex; Cr, creatine; NAA, N-acetylaspartate; Cho, choline; Ins, myo-inositol BCVA, Best-corrected visual acuity.

*P < 0.05.

## Discussion

This study investigated the metabolic changes in the V1 in blind patients with end-stage POAG using single-voxel ^1^H-MRS. The results showed that statistically increased Glx was detected in the V1 of end-stage POAG patients, which provided evidence of metabolic abnormalities in the V1. Furthermore, the Glx/Cr ratio in the POAG patients was significantly correlated with the mfERG, suggesting that metabolic alterations in the V1 might partly correlate with residual retinal function in blind subjects with end-stage POAG.

Glx is a critical neurotransmitter system in the mammalian brain, with peak concentrations in the cerebral cortex and hippocampus. Glutamate, the primary excitatory neurotransmitter, is released during synaptic activity and rapidly cleared through neuronal and glial transporters to prevent excitotoxicity (Zhou and Danbolt, 2014; Shen et al., 1999). Following uptake, glial cells convert glutamate to glutamine, sustaining the Glx cycle for neurotransmitter recycling and ammonia detoxification (Moreno et al., 2005). Pathologically, elevated Glx levels are observed in ischemia, hypoxia, and hepatic encephalopathy, reflecting disrupted glutamate homeostasis (Ott et al., 1993). Notably, Glx dysregulation has emerged as a critical contributor to neurodegeneration in glaucoma. Compromised axonal transport due to elevated intraocular pressure or hypoperfusion disrupts the retrograde delivery of neurotrophic factors to retinal ganglion cells while simultaneously promoting the accumulation of excitotoxins, including glutamate, in the extracellular matrix (Grieb and Rejdak, 2002). The excess extracellular Glx exacerbates oxidative stress and hyperactivates N-methyl-D-aspartate receptors, triggering calcium influx that activates calpain, induces nitric oxide synthase, and disrupts mitochondrial function, ultimately driving apoptosis through imbalanced pro-apoptotic and anti-apoptotic gene regulation (Luthra et al., 2005; Yenice et al., 2008). Furthermore, oxidative stress in glaucomatous tissues, evidenced by elevated reactive oxygen species and oxidative DNA damage in the trabecular meshwork and vitreous humor, synergizes with glutamate excitotoxicity to amplify neuronal injury (Chang and Goldberg, 2012).

Glutamate excitotoxicity caused by excessive glutamate was observed in both anterior and posterior visual pathways in glaucoma, such as the optic nerve head, the lateral geniculate body, and the V1 (Okuno et al., 2006; Doganay et al., 2012; Ho et al., 2015). It agreed with our prior ^1^H-MRS study, which demonstrated the excessive Glx within V1 in early glaucoma patients (Guo et al., 2018). Similarly, the current study showed that statistically increased Glx was detected in the V1 of the blind patients of end-stage POAG patients. In glaucoma pathogenesis, reduced ascending afferent input to the V1 secondary to visual impairment has been hypothesized to impair glutamate uptake and/or disrupt its metabolic homeostasis, resulting in pathological accumulation of the neurotransmitter within the extracellular compartment (Chan et al., 2009). While elevated Glx/Cr may reflect increased extracellular glutamate contributing to excitotoxicity, we acknowledge that ^1^H-MRS at 3T cannot spectrally separate glutamate from glutamine. Thus, alternative explanations must be considered: altered glutamate-glutamine cycling, where glial-derived glutamine sustains neuronal glutamate pools; compensatory upregulation of glutamatergic neurotransmission in response to deafferentation; and astrocyte-mediated detoxification of excess glutamate *via* glutamine synthesis. Although glutamate-dominated excitotoxicity remains plausible in glaucoma pathogenesis, our data cannot exclude significant contributions from glutamine.

Interestingly, we also found a relationship between the Glx/Cr ratio in V1 and the N1-wave latency of the mfERG in end-stage POAG patients. The N1 wave represents phototransduction and signal transmission from photoreceptors to bipolar cells. In glaucoma, glutamate concentration dynamics modulated by altered reuptake efficiency and metabolic processing may induce adaptive cortical reorganization within the V1, potentially driving compensatory plasticity across posterior visual pathways (McCoy et al., 2009). Emerging evidence underscores retinocortical functional coupling in retinal degenerative diseases, demonstrating enhanced ERG-fMRI correlations in Stargardt disease (Melillo et al., 2018). Our prior work established analogous retinocortical interplay in end-stage glaucoma, where blood-oxygen-level-dependent functional MRI (BOLD-fMRI) beta values in foveal retinotopic areas correlated with mfERG P-wave amplitudes (Zhu et al., 2022). The present ^1^H-MRS findings extend this paradigm, revealing metabolic glutamate signatures in V1 that parallel residual retinal electrophysiology, independent of structural degeneration metrics. The moderate-to-strong inverse correlation between Glx/Cr and retinal function (effect size ρ = −0.68), though uncorrected, aligns mechanistically with retinocortical coupling literature. We emphasize this as a preliminary finding warranting validation in larger cohorts. These converging multimodal insights position metabolic-functional cortical profiling as a novel biomarker framework for low-vision rehabilitation, transcending traditional structural assessments of retinal nerve fiber layers.

Ins, a cyclic polyol predominantly synthesized by cerebral glial cells, serves as a critical organic osmolyte mediating brain osmoregulation (Harris et al., 2011). Astrocytes dynamically adjust Ins levels through transmembrane transport to counterbalance extracellular osmotic fluctuations (Elberling et al., 2003). During glutamate-induced osmotic stress, intracellular Ins accumulation occurs concomitantly with decreased extracellular pool concentrations (Razek et al., 2014). Our prior ^1^H-MRS findings demonstrated reduced Ins levels in early glaucoma, correlating with elevated extracellular glutamate concentrations in the V1 (Guo et al., 2018). Paradoxically, Bernabeu et al. reported Ins elevation in V1 of blind subjects with retinal and optic nerve disorders despite unchanged Glx levels (Bernabeu et al., 2009). While Ins participates in phosphatidylinositol secondary messenger systems and neuroplasticity mechanisms (Jansonius, 2005), our study revealed no significant Ins alterations in V1 of end-stage glaucoma patients with blindness. These divergent observations suggest disease category-specific and duration-dependent modulation of Ins homeostasis during glutamate-mediated cortical adaptation processes. Our null Ins finding aligns with glaucoma-specific astrocyte pathophysiology: Early-stage IOP stress: Transient Ins depletion occurs as Müller cells consume inositol for osmotic compensation against glutamate excitotoxicity; End-stage glial exhaustion: Chronic oxidative stress inactivates sodium-myo-inositol cotransporters (SMIT1), limiting Ins accumulation despite persistent osmotic imbalance. This parallels retinal findings where advanced glaucoma shows reduced Müller cell density.

NAA, a neuron-specific metabolite maintaining high cerebral concentrations, is an established biomarker of neuronal degeneration (Gujar et al., 2005). Cho, primarily comprising phosphorylated derivatives, reflects membrane turnover processes, including myelination dynamics (Chan et al., 2009; Zhang et al., 2013). Longitudinal studies have documented progressive NAA and Cho reductions along the anterior visual pathway in early and advanced glaucoma by using MRS (Pang et al., 2024; Zhang et al., 2013). In contrast, the current study revealed preserved NAA and Cho levels within V1, similar to the previous ^1^H-MRS study (Bernabeu et al., 2009; Guo et al., 2018; Sidek et al., 2016). The metabolic preservation in end-stage glaucoma may reflect temporal dynamics of metabolic compensation, where early-phase compensatory upregulation of membrane turnover and mitochondrial function may lead to stabilized metabolic equilibrium at terminal disease stages. Concurrently, experience-dependent neuroplasticity may engage compensatory metabolic networks, particularly tactile/auditory cross-modal reorganization, and Müller glia-driven glutamate cycling, to bypass neurodegeneration-related metabolic stress. The preserved NAA/Cr in V1 contrasts sharply with NAA reductions in anterior visual structures and mirrors retinal ganglion cell (RGC) apoptosis timelines in glaucoma. While RGC loss propagates anterograde degeneration to the lateral geniculate nucleus (LGN) within months, V1 neurons demonstrate remarkable resistance to trans-synaptic degeneration. Stable Cho/Cr reflects phospholipid equilibrium unique to glaucoma’s posterior visual pathway. Unlike anterior optic radiations, where Cho reductions signify Wallerian degeneration, V1 exhibits **Müller glia-driven membrane recycling**. Activated retinal Müller cells release phosphatidylcholine precursors that reach the cortex *via* a disrupted blood-brain barrier in glaucoma, compensating for axonal membrane loss.

Similar to auditory cortex reorganization in deafness (Bola et al., 2017a) and somatosensory cortex changes in spinal injury (Höller et al., 2017), glaucoma-induced visual deprivation drives cross-modal takeover of V1. However, glaucoma uniquely combines retrograde neurodegeneration with anterograde metabolic compensation, creating a distinct “disconnection-reorganization” paradigm. Glx elevation may facilitate cross-modal reorganization, analogous to blind individuals repurposing V1 for Braille processing (Bola et al., 2017b). Persistent Glx could sustain V1 excitability for non-visual inputs, explaining neuronal preservation despite blindness.

This investigation has several limitations requiring consideration. First, the modest cohort size (n = 11 per group) constrains statistical power for detecting subtle metabolic changes and precludes stratification by critical variables like blindness duration or residual retinal function profiles. Second, systemic absorption of topical agents (e.g., β-blockers, prostaglandin analogs) might alter cerebral blood flow, potentially confounding metabolic measurements. We cannot exclude vascular-mediated metabolic effects. Third, the uncorrected multiple comparisons increase the false-positive risk. Our small sample remains underpowered for stringent multiplicity adjustments. Future studies should pre-specify primary outcomes and employ hierarchical testing frameworks. Finally, 3T ^1^H-MRS cannot separate glutamate and glutamine signals; therefore*, the observed Glx elevation is not due solely to increased glutamate.* And single-voxel MRS cannot resolve layer-specific gradients. Future ultrahigh-field (7T) ^1^H-MRS studies with improved spectral resolution are warranted to dissect glutamate and glutamine contributions and clarify the neurotoxic and compensatory roles of Glx elevation.

In conclusion, our findings demonstrate that end-stage POAG with profound blindness exhibits distinct glutamate-mediated metabolic adaptation within V1, characterized by elevated Glx levels alongside preserved concentrations of NAA, Cho, and Ins. This metabolic profile suggests active neuroglial compensation, potentially involving changes in glutamine levels or altered glutamate-glutamine cycling, that maintains cortical viability despite chronic deafferentation. Critically, the correlation between V1 Glx/Cr ratios and residual retinal responses challenges the paradigm of complete anteroposterior visual pathway disconnection in end-stage disease. The persistence of retinocortical signaling, evidenced by Glx/Cr-mfERG coupling, provides a neurochemical basis for three targeted rehabilitation approaches: Glutamate-modulated sensory substitution devices that convert visual inputs to auditory/tactile signals synchronized with residual ERG activity**;** retinotopic-specific neuromodulation amplifying glutamatergic pathways in V1; personalized rehabilitation timing where MRS-detected Glx elevation identifies optimal neuroplastic windows for intervention.

## Data Availability

The original contributions presented in the study are included in the article/supplementary material, further inquiries can be directed to the corresponding author.
